# Human periodontal ligament stem cells promote oral ulcer healing in rats through modulation of TGF-β1/smad signaling

**DOI:** 10.3389/fcell.2026.1824785

**Published:** 2026-08-28

**Authors:** Xiang Cai, Guoguo Jin, Wei Wang, Lingyue Wu, Kingsing Tam, Xiaotian Wang, Yue Wang, Huajian Tian, Qi Xiang, Zhiying Zhou

**Affiliations:** 1 State Key Laboratory of Bioactive Molecules and Druggability Assessment, Jinan University, Guangzhou, China; 2 Institute of Biomedicine and Guangdong Provincial Key Laboratory of Bioengineering Medicine, Jinan University, Guangzhou, China; 3 Biopharmaceutical R&D Center of Jinan University, Guangzhou, China; 4 School of Stomatology, Jinan University, Guangzhou, China; 5 Department of Orthodontics, The First Affiliated Hospital of Jinan University, Guangzhou, China; 6 Gannan Health Vocational College, Ganzhou, China

**Keywords:** fibrosis, hPDLSCs, inflammation, oral ulcer healing, oral ulcers, TGF-β1

## Abstract

**Background:**

Oral ulcers (OU) often present with prolonged healing, recurrent episodes, and scar formation, posing challenges for clinical management. Human periodontal ligament stem cells (hPDLSCs) have shown potential in oral tissue repair, but further research is needed to clarify their mechanism of action in OU healing. This study aims to elucidate the molecular mechanisms by which hPDLSCs promote oral ulcer healing.

**Method:**

To identify key regulatory genes, the OU-associated microarray dataset GSE37265 was integrated with hPDLSC genomic data for differential expression analysis. Subsequently, Weighted Gene Co-expression Network Analysis (WGCNA) was used to identify functional modules associated with OU healing. *In vivo*, hPDLSCs were locally administered into a rat ulcer model, and therapeutic efficacy was assessed by ulcer closure rates and histological evaluation (HE and Masson’s trichrome staining). Furthermore, RNA-sequencing (RNA-seq) was performed on oral mucosal tissues to delineate the underlying molecular landscape and critical signaling pathways. The involvement of the TGF-β signaling pathway was confirmed by real-time quantitative PCR (RT-qPCR) and Western blotting (WB) analyses.

**Results:**

Bioinformatics analysis identified 92 key genes in hPDLSCs-mediated treatment of OU, highlighting the central role of the TGF-β1/Smad pathway. As shown by the animal studies, hPDLSCs therapy increased the healing rate to 97% by day 8 (*vs*. 70% in the model). Furthermore, the therapy significantly reduced inflammatory cell infiltration and abnormal collagen deposition while promoting regular collagen arrangement. Transcriptomic and molecular experiments further showed that hPDLSCs simultaneously inhibit TGF-β1/Smad and extracellular signal-regulated kinase (ERK) signaling pathways, thereby alleviating inflammatory responses and suppressing mucosal fibrosis.

**Conclusion:**

In this study, we reveal a novel role for hPDLSCs in promoting oral ulcer healing. The findings indicate that hPDLSCs suppress inflammation and fibrosis via the TGF-β1/Smad pathway, offering a promising therapeutic strategy for OU and other fibrotic conditions.

## 1 Introduction

Oral ulcers (OU) are among the most common lesions of the oral mucosa, characterized by full-thickness epithelial loss and exposure of the underlying connective tissue (Babu et al., 2017; Yogarajah and Setterfield, 2021). These lesions are typically accompanied by intense pain, causing difficulties in eating and speaking, severely impairing patients’ quality of life (Stoopler et al., 2024).

Chronic OU are frequently driven by persistent inflammatory dysregulation and excessive collagen deposition. Over time, this pathology progresses to irreversible fibrosis and scar formation, impairing tissue function (Griffin et al., 2022). Such remodeling not only degrades mucosal elasticity and regenerative potential but, in severe cases, complicates orthodontic outcomes and reduces patient compliance (Pan et al., 2024). Standard interventions—ranging from topical corticosteroids, anti-inflammatories, and antibiotics to surgical debridement—are largely palliative (Fourie and Boy, 2016). While they alleviate symptoms, prolonged use risks local irritation, drug resistance, and opportunistic fungal infections (Vigneswaran and Muller, 2024). Existing therapies fail to achieve optimal tissue regeneration, leaving a significant unmet need in dental medicine. Regenerative medicine offers a new frontier for restoring tissue architecture, with stem cell-based therapies at the forefront. hPDLSCs are particularly promising candidates for mucosal repair, given their multipotency, immunomodulatory properties, and ready accessibility from extracted teeth (Wen et al., 2024). Although preclinical studies suggest hPDLSCs enhance angiogenesis and reduce inflammatory cytokines in oral wounds (Zhang et al., 2020; Wen et al., 2024), it remains unclear whether—and how—they can simultaneously suppress fibrosis and inflammation through coordinated pathway modulation. As a master regulator of tissue repair, the TGF-β1/Smad signaling pathway plays a dual role in wound healing. Transient TGF-β1 activation promotes epithelial regeneration, yet sustained signaling triggers fibroblast-to-myofibroblast transition and pathological collagen accumulation (Meng et al., 2016; Hu et al., 2018). Recent evidence indicates that stem cells can modulate TGF-β1 activity in a spatiotemporal manner to favor regeneration (Kim et al., 2018). Additionally, crosstalk between TGF-β1/Smad and the ERK signaling pathway appears to amplify fibrotic cascades, though strategies to disrupt this interaction are understudied (Ye and Hu, 2021). We hypothesized that hPDLSCs might modulate TGF-β1/Smad signaling, attenuating its sustained pathological activation while preserving acute regenerative functions. To systematically investigate the mechanisms of hPDLSCs, we employed an acute oral ulcer model. This controlled setting reduces confounders from persistent pathology, allowing isolation of the regenerative and anti-fibrotic functions of transplanted cells. We hypothesize that hPDLSCs orchestrate oral ulcer regeneration by fine-tuning TGF-β1/Smad signaling and its interplay with ERK, thereby simultaneously suppressing inflammatory cascades and blocking fibrosis. By integrating clinical data mining with a rat model, how do hPDLSCs regulate TGF-β1/Smad-ERK crosstalk to achieve these dual effects? The insights gained from this acute setting provide a mechanistic foundation for future applications in the complex clinical context of chronic OU.

## 2 Materials and methods

### 2.1 Subsection acquisition and screening of target OU-related and hPDLSCs-related genes

The GSE37265 dataset was obtained from the GEO database (www.ncbi.nlm.nih.gov/geo/) using the keywords “OU”, “oral mucositis”, and “aphthous ulcers”. The study used 28 samples, comprising 14 ulcerated and 14 normal mucosa samples, from patients enrolled in the OU cohort. These samples were generated on the GPL570 platform. To identify hPDLSC-associated genes, the GeneCards database (https://www.genecards.org/) was interrogated, as it provides functional annotations for known and predicted human genes. Genes with a relevance score of at least 7 were selected, yielding 1,880 candidates. A relevance score ≥7 was chosen based on the platform’s recommendation for high-confidence gene associations, balancing sensitivity and specificity.

### 2.2 Bioinformatics analysis

Bioinformatics analyses were conducted as previously described (Zhao et al., 2023). Differentially Expressed Genes (DEGs) in GSE37265 were identified using the R “limma” package (|log FC| > 1, *p* < 0.05) and visualized with “ggplot2” and “pheatmap.” A co-expression network was built with WGCNA, including sample clustering, soft-thresholding (soft-thresholding power β = 6, chosen as the lowest power that reached a scale-free topology fit index R^2^ > 0.85), dissimilarity calculation, and dynamic hybrid cutting for module detection (min Module Size = 30, merge Cut Height = 0.25). Intersection genes were obtained by overlapping hPDLSCs-related genes, key WGCNA module genes, and DEGs using the “venn” R package. This integrative approach was designed to prioritize genes that are both dysregulated in OU (DEGs), co-expressed with clinical traits (turquoise module), and functionally relevant to hPDLSCs, thereby narrowing down candidates most likely to mediate therapeutic effects. GO and KEGG enrichment analyses were performed with “cluster Profiler” (*p* < 0.05). A PPI network was constructed from the STRING database (*Homo sapiens*) and visualized in Cytoscape (version 3.6.0).

### 2.3 Isolation and cultivation of hPDLSCs

hPDLSCs were derived from the root surface tissues of ten healthy orthodontic patients (five males and five females aged 18–20 years) who were recruited from the First Affiliated Hospital of Jinan University. Cell isolation and culture were performed according to the established protocol (Huang et al., 2021), and cells were expanded to passage 2. Stem cell characterization was performed by flow cytometry for surface markers (positive: CD90, CD105, CD166, CD44; negative: CD34, CD45, HLA-DR) and by multilineage differentiation assays. Osteogenic and chondrogenic differentiation were induced with lineage-specific media and verified by Alizarin Red S and Alcian Blue staining, respectively.

### 2.4 OU model establishment and grouping

Twenty-eight male Sprague-Dawley rats (7–8 weeks old; 200–220 g) from the Guangdong Medical Laboratory Animal Center were housed under specific pathogen-free conditions at 22 °C–25 °C, 60%–65% humidity, with a 12-h light/dark cycle. After 1 week of acclimatization, oral ulcers were induced by placing a 5-mm-diameter filter paper disc saturated with 70% acetic acid on the oral mucosa for 3 min. Rats with ulcers were randomly assigned to treatment and control groups (n = 14 each) via a random number table. Each group was subdivided into two observation points (Day 4 and Day 8; n = 7), with three rats per point for histology and four for molecular analyses (RNA and protein extraction). Fourteen SD rats were treated by submucosal injection of hPDLSCs (1 × 10^6^) suspended in 200 μL of phosphate-buffered saline (PBS) at five points around the ulcer. The cell dose was selected based on previous studies (Ryu et al., 2024). Fourteen additional rats received PBS only and served as the control group. Personnel administering the treatments were aware of the group assignments because of the procedure’s design. All injections were administered on day 1, the day after ulcer induction. Wound areas were photographed on Days 2, 4, 6, and 8. To minimize assessment bias, these measurements, along with all subsequent histopathological evaluations, were performed by investigators who were fully blinded to group assignments. Biochemical measurements were performed in triplicate to ensure technical reliability. It is important to note that certain variables, such as treatment order, measurement sequence, and cage placement, were not strictly controlled in this study. Rats were euthanized by cervical dislocation under anesthesia with 3% pentobarbital sodium (3 mg/100 g, Sinopharm, China) given intraperitoneally. Buccal mucosal tissues were collected on Days 4 and 8. The tissues were fixed in 4% paraformaldehyde for 24 h, then dehydrated through an ethanol gradient, cleared with xylene, and embedded in paraffin. Sections of 3 µm thickness were prepared and stained with hematoxylin-eosin (HE) or Masson’s trichrome for histological analysis using a light microscope (Easyscan, Motic®, China).

### 2.5 Fluorescence *in situ* hybridization (FISH)

FISH was performed as previously described (Li et al., 2024). A custom fluorescein-labeled probe targeting PLAP-1 mRNA (5′-GGG​ACA​GA-TAC​AGC​CTT​CGC​AAC​T-3'; Servicebio, China) was used at 500 nM.

### 2.6 RNA-seq

Total RNA was extracted from ulcerated oral mucosal tissues of rats using TRIzol reagent (Thermo Fisher Scientific, United States of America) according to the manufacturer’s protocol. The quality of RNA was evaluated with an Agilent 2,100 Bioanalyzer. Library construction and sequencing were performed on an Illumina NovaSeq 6,000 platform. Raw reads were quality-trimmed using fastp (v0.23.2), and aligned to the rat reference genome (rn6) using HISAT2 (v2.2.1). PCA and differential expression analysis (DESeq2 v1.36.0) were conducted in R, with thresholds of | log_2_ FC | > 1 and adjusted p-value (Benjamini–Hochberg) < 0.05, and functional enrichment (GO, KEGG) and correlation analyses were performed using the OmicShare platform (www.omicsmart.com).

### 2.7 RT-qPCR

RT-qPCR was performed as previously described (Zhao et al., 2023). The primer sequences were as follows:

GAPDH (F: GCA​AGT​TCA​ACG​GCA​CAG; R: CGC​CAG​TAG​ACT​CCA​CGA​C); ERK (F: GGT​AGA​CGG​TTC​TGG​AAT​GGA​AGG; R: GTCAGGGAAAATGGGGTG GG); TGF-β1 (F: CAC​CAT​CCA​TGA​CAT​GAA​CC; R: TCA​TGT​TGG​ACA​ACT​GCT​CC); CTGF (F: CAG​TTG​GCT​CGC​ATC​ATA​GTT​G; R: GTG​TGT​GAT​GAG​CCC​AAG​GA); IL-6 (F: GGC​CCT​TGC​TTT​CTC​TTC​G; R: ATA​ATA​AAG​TTT​TGA​TTA​TGT).

### 2.8 Western blot

Western blot was performed as described previously (Li et al., 2024). Primary antibodies used included GAPDH (1:1,000; #BS-10900R, Bioss, China), Tubulin (1:1,000; #2144, CST, United States of America), TGF-β1 (1:1,000; #3711, CST, United States of America), CTGF (1:1,000; #HY-P81103,MCE, China), Smad2/3 (1:1,000; #HY-P80893, MCE, China), p-Smad2/3 (1:1,000; #ER64981, Huabio, China), ERK (1:1,000; #ET1601-29, Huabio, China), and p-ERK (1:1,000; #ET1610-13, Huabio, China).

### 2.9 Cytokines detection

Human periodontal ligament stem cells (hPDLSCs) and human oral keratinocytes (HOK) were seeded separately in 6-well plates at 1 × 10^5^ cells/well and cultured in serum-free medium for 48 h. Culture supernatants were collected and centrifuged at 1,500 rpm for 10 min at 4 °C to remove debris. Concentrations of multiple cytokines (including IL-8, IL-2, IL-10, IL-1β, etc.) in the supernatants were quantified using the Aptplex™ Human Cytokine 2-Plex Panel(C) (Elabscience, Wuhan, China; MPD001), a multiplex bead-based flow cytometry immunoassay. The assay employs fluorescently labeled magnetic microspheres coated with capture antibodies, biotinylated detection antibodies, and PE-labeled streptavidin. Briefly, 50 μL of sample or standard was mixed with antibody-coupled beads and incubated for 2.5 h at room temperature with shaking. After washing, the beads were resuspended and acquired on a Beckman Coulter Gallios flow cytometer (Beckman Coulter, Brea, CA, United States of America) equipped with two lasers. Data were analyzed using the instrument’s software (Kaluza), and cytokine concentrations were calculated from standard curves. Each sample was measured in triplicate. Data are presented as mean ± SD.

### 2.10 *In vitro* co-culture and IL-8 blockade assays

HOK were cultured in keratinocyte growth medium, and hPDLSCs were maintained as described. Rat neutrophils were freshly isolated from peripheral blood using a neutrophil isolation kit (Solarbio P9200, Beijing Solarbio Science and Technology Co., Ltd.) via density gradient centrifugation, followed by red blood cell lysis strictly according to the manufacturer’s instructions. For co-culture, HOK cells were seeded into the lower chambers of 6-well transwell plates (0.4 μm pore size) at 2 × 10^5^ cells per well; hPDLSCs were seeded into the upper inserts at 1 × 10^5^ cells per insert; and freshly isolated rat neutrophils were added to the lower chamber at 2 × 10^5^ cells per well (HOK:neutrophil = 1:1). Five experimental groups were established: ① HOK alone; ② hPDLSCs + HOK; ③ HOK + neutrophils; ④ hPDLSCs + HOK + neutrophils; and ⑤ hPDLSCs + HOK + neutrophils + IL-8 neutralizing antibody (5 μg/mL). After 48 h of co-culture, total protein was extracted from the HOK cells in the lower chamber, and protein concentrations were determined using a BCA assay. Equal amounts of protein were then subjected to Western blot analysis for TGF-β1.

### 2.11 Statistical analysis

Statistical analyses were performed using GraphPad Prism 9.0. All data are presented as the mean ± standard deviation (SD) from a minimum of three independent experiments performed in triplicate. The analysis used an unpaired Student’s t-test to compare two groups. Multi-group comparisons were performed using one-way ANOVA with Tukey’s *post hoc* test. Longitudinal healing data were analyzed by two-way repeated measures ANOVA with Bonferroni correction. Statistical significance was defined as *p < 0.05, **p < 0.01, ***p < 0.001 and ****p < 0.0001 compared to the control group.

## 3 Results

### 3.1 WGCNA revealed a pivotal Co-expression module associated with oral ulcer

**FIGURE 1:**
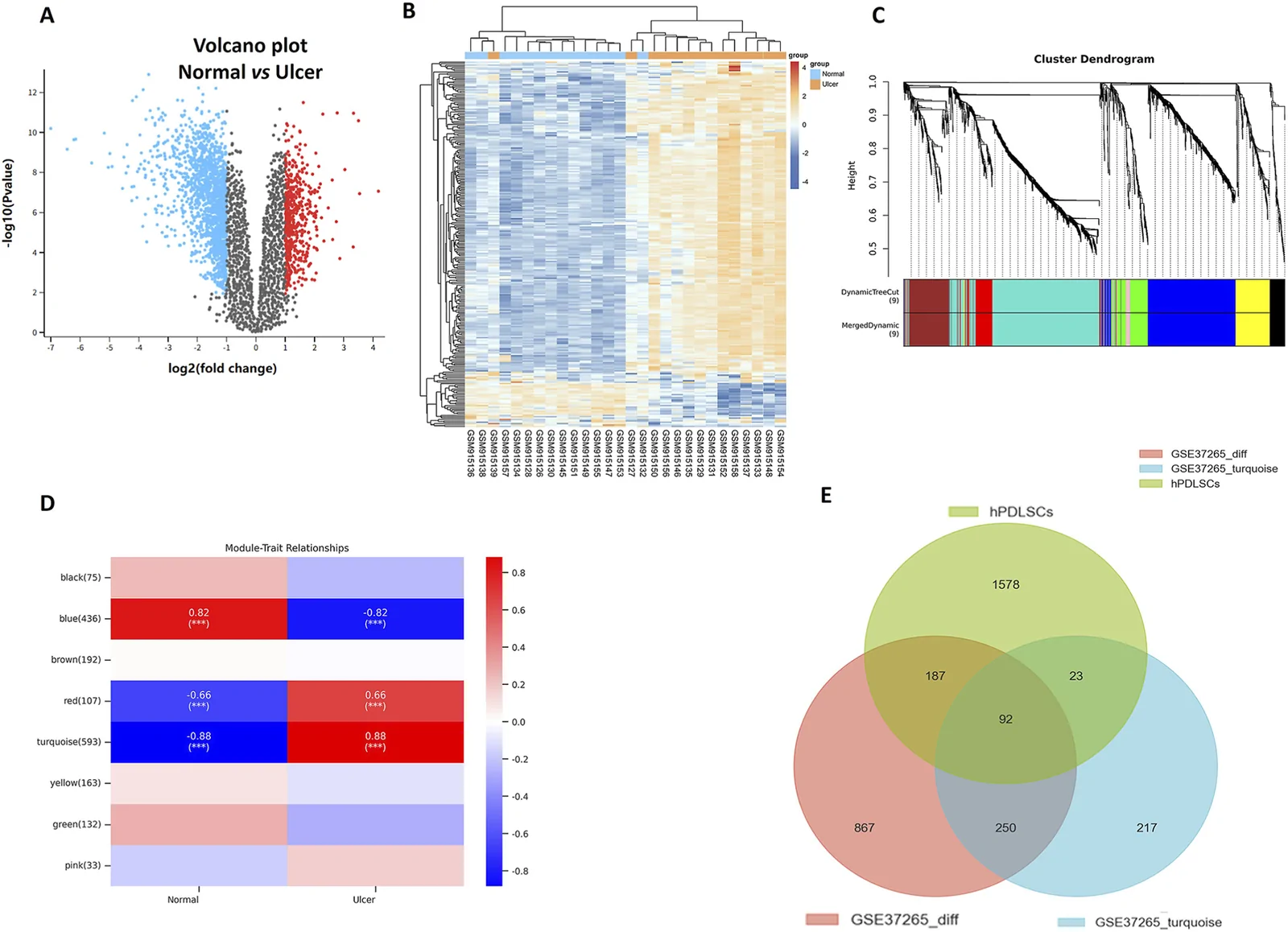
Differential analysis and WGCNA identified key genes related to OU healing. **(A)** Volcano plot of GSE37265; gray: no significant change, red: upregulated, blue: downregulated. **(B)** Heatmap of all DEGs. **(C)** WGCNA gene co-expression dendrogram; colors denote modules. **(D)** Module-trait correlation heatmap with coefficients and p-values. **(E)** Venn diagram showing DEGs, turquoise module genes, and hPDLSCs-related genes.

First, differential analyses were conducted on the GSE37265 dataset to identify key genes involved in OU. 1,315 DEGs were selected, including 328 upregulated and 987 downregulated (volcano plot, Figure 1A). The clustered heatmap (Figure 1B) shows the expression patterns of all DEGs, indicating that samples within the same group (either Normal or OU) are highly consistent, whereas the two groups differ significantly. The OU group had a higher number of downregulated DEGs, suggesting that these genes may play a key role in OU pathogenesis.

To identify core genes associated with OU disease, we used WGCNA to analyze GSE37265 and performed functional enrichment analyses to understand their biological roles. Our analysis identified nine co-expression modules in the OU expression profile; however, the gray module was considered uninformative because its genes could not be assigned to specific functional categories (Figure 1C). After removing the gray module, eight distinct co-expression modules were identified, comprising the black (75 genes), blue (463 genes), brown (192 genes), red (107 genes), turquoise (593 genes), yellow (163 genes), green (132 genes), and pink (33 genes) modules. Correlation analysis between module eigengenes and clinical traits showed that the turquoise module had the strongest positive association with the disease (correlation coefficient = 0.88). As a result, the 593 genes within the turquoise module were identified as core genes related to OU (Figure 1D).

Finally, to better identify the key genes involved in hPDLSC treatment of OU, the DEGs from the GSE37265 microarray dataset, the Turquoise module genes, and the hPDLSC-related genes were intersected, yielding 92 overlapping genes for subsequent functional analysis (Figure 1E).

### 3.2 TGF-β1 signaling pathway as central to inflammation and fibrosis in oral ulcer healing

**FIGURE 2:**
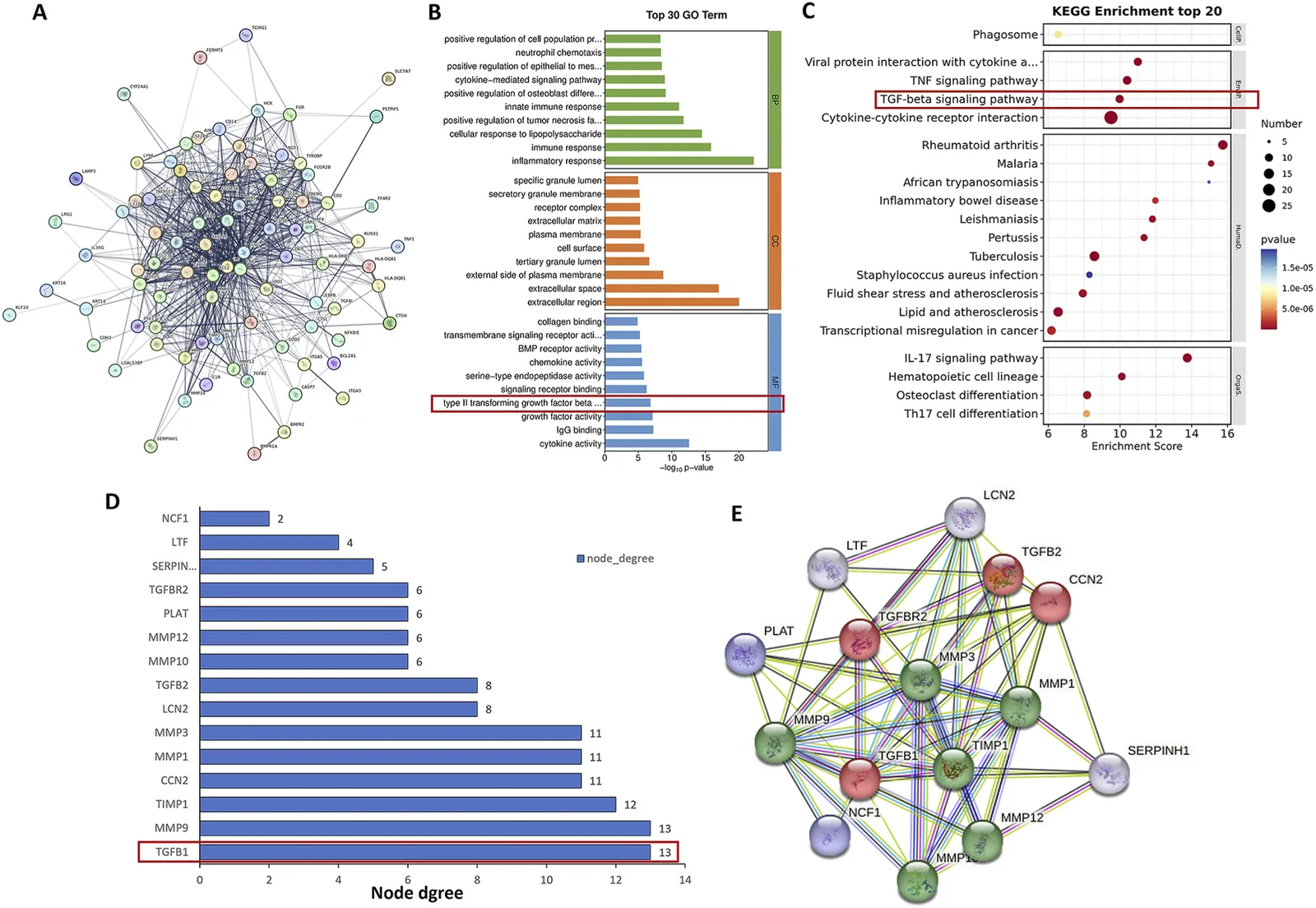
The TGF-β1 pathway may influence hPDLSCs treatment of OU. **(A)** PPI network of key genes. **(B)** GO analysis at BP, CC, and MF levels. **(C)** KEGG pathway enrichment. **(D)** Gene degree values. **(E)** TGFB1 expression in key genes.

Reducing inflammation is a crucial phase in OU healing. To investigate this, an inflammation-related gene set was identified using the GSEA database. A STRING analysis was performed to create a PPI network map highlighting inflammation and key genes involved in the hPDLSCs treatment of OU (Figure 2A). Building on previous research, the molecular functions (MFs) of these candidate genes were examined in detail, and GO function, along with KEGG pathway analyses, were conducted on the candidate genes.

To characterize the biological roles of the identified candidate genes, we performed Gene Ontology (GO) functional annotation. The results revealed that, in the Biological Process (BP) domain, candidate genes were predominantly involved in orchestrating inflammatory and immune responses, as well as in cytokine-mediated signaling. Cellular Component (CC) analysis indicated a significant localization within the extracellular space, cell surface, and receptor complexes. Regarding Molecular Function (MF), these genes primarily exhibited activities related to cytokine binding, growth factor regulation, and collagen interaction (Figure 2B).

Consistent with the GO findings, KEGG pathway enrichment highlighted the involvement of these genes in cytokine-cytokine receptor interaction, TGF-β signaling, TNF signaling, and Th17 cell differentiation (Figure 2C). These bioinformatic signatures suggest that the candidate genes act as key extracellular modulators of the immunological landscape during disease progression.

A PPI network was modeled to dissect the connectivity between DEGs and inflammatory mediators. TGFB1 was identified as a high-degree hub protein, demonstrating extensive interactions with multiple neighbors (Figure 2D). Beyond its established role in immunomodulation, TGFB1 is a pleiotropic factor essential for angiogenesis, bone remodeling, and tissue repair.

Sub-network analysis specifically focused on collagen metabolism revealed that TGFB1 maintains high connectivity with TGFBR2, TGFB2, CCN2, MMP3, and MMP9 (Figure 2E). These nodes are critical regulators of the extracellular matrix (ECM) turnover, balancing collagen synthesis and degradation—a prerequisite for oral ulcer (OU) recovery. The convergence of TGFB1, TGFBR2, and CCN2 further highlights the role of the TGF-β axis in hPDLSC-mediated therapy.

The TGF-β1 signaling pathway is a complex, multifunctional network that controls various processes such as cell growth, immune regulation, tissue repair, and fibrosis (Lichtman et al., 2016). Under specific conditions, such as tissue injury and inflammatory response, TGF-β1 is activated and binds to type II receptors on the cell membrane, thereby activating downstream Smads and regulating the expression of target genes (Aashaq et al., 2022). These target genes may include those involved in cell proliferation, differentiation, apoptosis, and extracellular matrix production. Additionally, TGF-β has been linked to the regulation of proliferation, differentiation, migration, and survival in various cell types, particularly fibroblasts, thereby influencing fibrosis and tissue repair (Meng et al., 2016; Kim et al., 2018). Based on our study of the role of hPDLSCs in the treatment of OU, we hypothesized that hPDLSCs may attenuate the inflammatory response and regulate fibrosis by modulating the TGF-β1 signaling pathway to promote healing in OU.

### 3.3 hPDLSCs transplantation improves oral ulcer healing in rats

**FIGURE 3:**
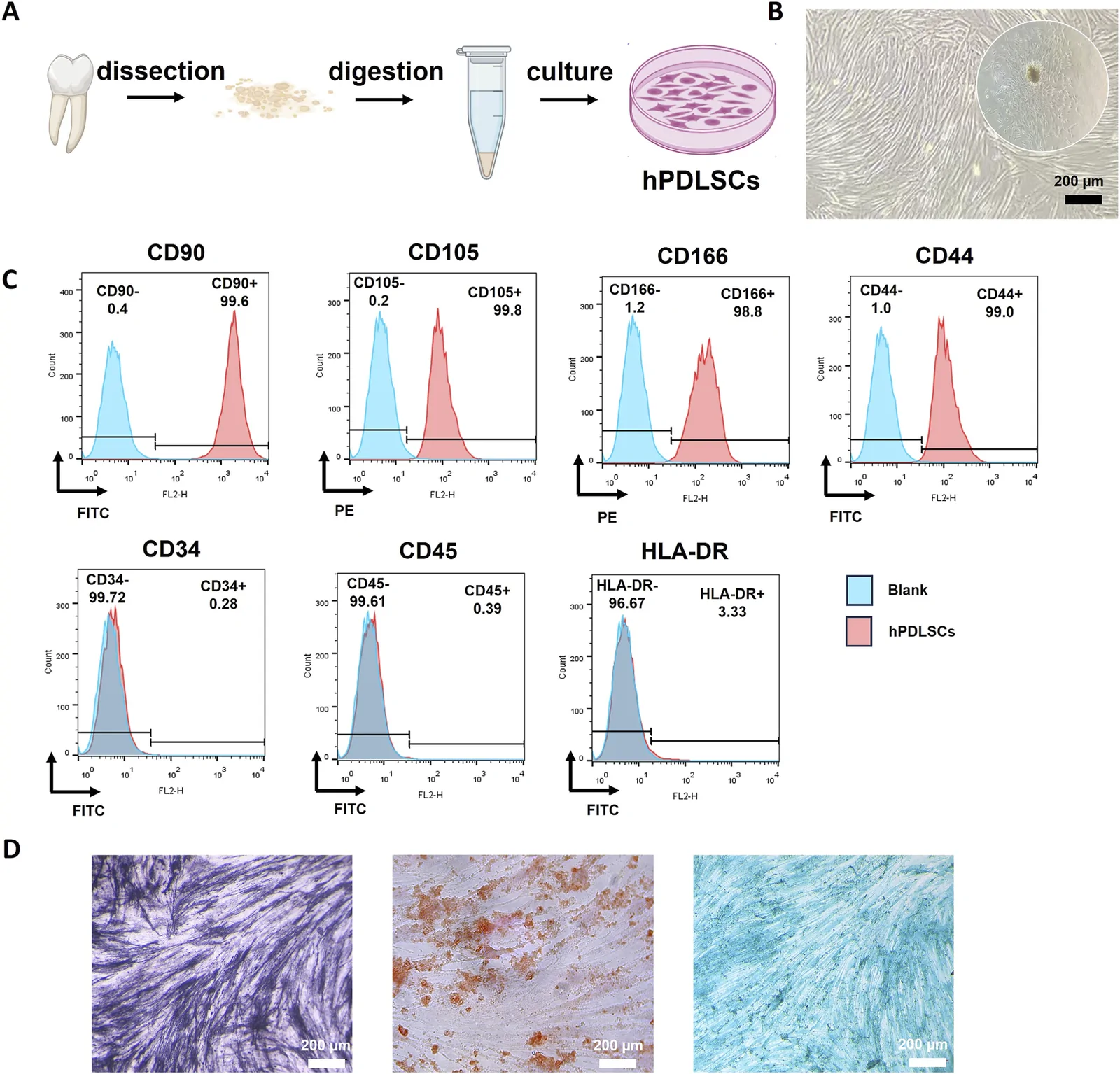
Isolation, characterization, and multilineage differentiation of hPDLSCs. **(A)** Schematic diagram of the extraction and culture procedure. **(B)** Light microscopy image of cultured hPDLSCs; The scale bar is 200 μm. **(C)** Flow cytometric analysis of surface marker expression, showing mesenchymal markers (CD90, CD105, CD166, CD44) and hematopoietic/endothelial markers (CD34, CD45, HLA-DR). **(D)** Multilineage differentiation capacity: ALP staining after 7 days of osteogenic induction; Alizarin red S staining after 21 days of osteogenic induction; Alcian blue staining after 21 days of chondrogenic induction. The scale bar is 200 μm.

Before *in vivo* experiments, hPDLSCs were isolated and cultured as illustrated (Figure 3A), and exhibited spindle-shaped morphology (Figure 3B). Flow cytometry showed high expression of CD90, CD105, CD166, and CD44, while being negative for CD34, CD45, and HLA-DR (Figure 3C). Multilineage differentiation was confirmed by ALP and Alizarin red S staining for osteogenesis, and Alcian blue staining for chondrogenesis (Figure 3D). These results confirm successful isolation and stemness maintenance of hPDLSCs.

**FIGURE 4:**
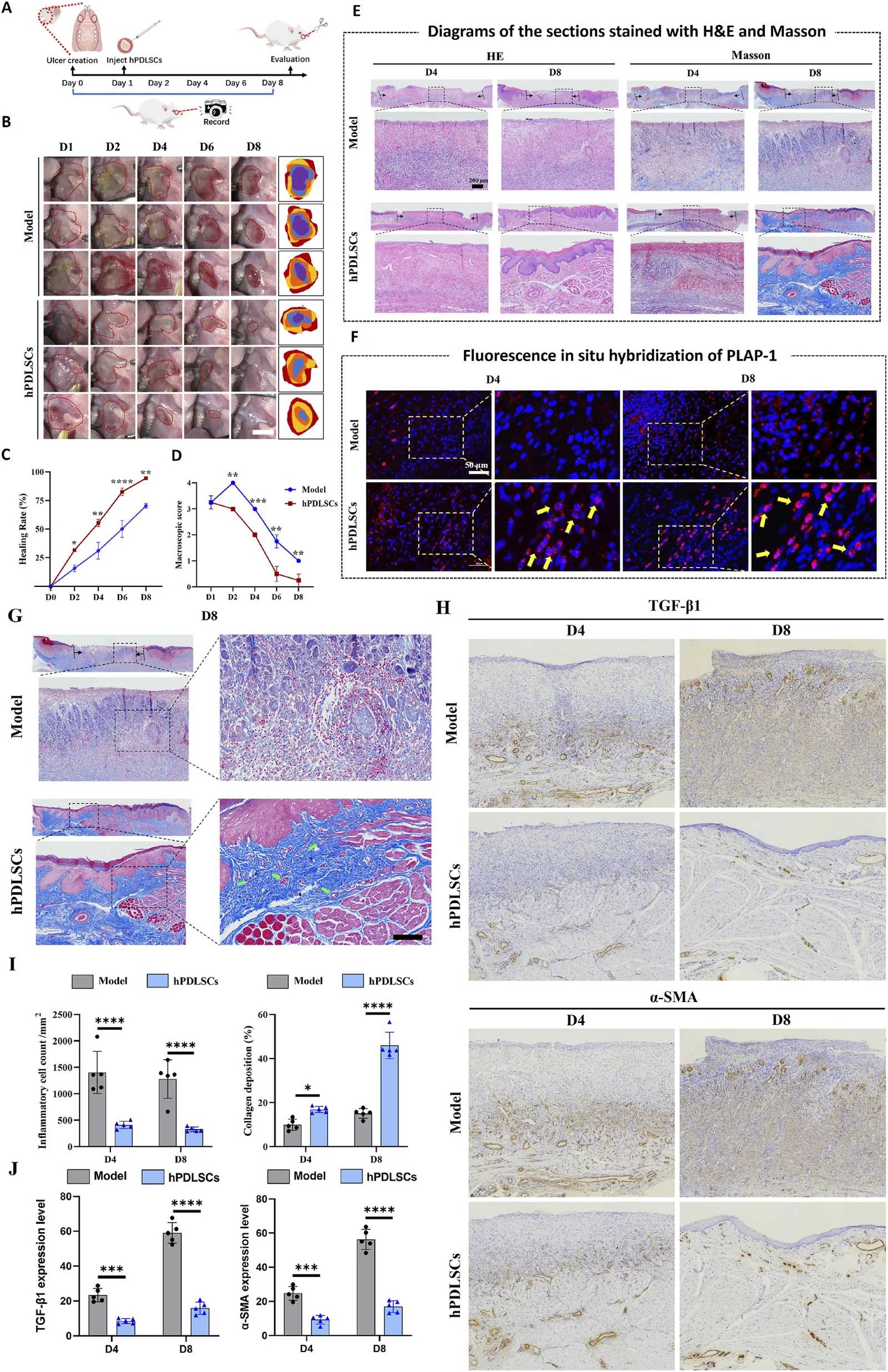
hPDLSCs promote ulcer healing in OU rats. **(A)** Schematic of experimental procedure. **(B)** Ulcer healing observation. **(C)** Healing rates for OU. **(D)** Macroscopic scores for OU severity. **(E)** HE and Masson staining of ulcerated oral mucosa tissues from hPDLSCs and model groups on Days 4 and 8 **(F)** FISH for PLAP-1. The red channel shows PLAP-1, blue shows DAPI, and yellow arrows point to PLAP-1-positive cells. Several are in the hPDLSCs group, but none in the model group. **(G)** Masson’s trichrome stain of oral ulcer shows green arrows pointing to neovascular capillaries; scale bar is 100 μm. **(H)** IHC for TGF-β1 and α-SMA on Days 4 and 8. The scale bar is 100 μm. **(I)** Quantitative analysis of inflammatory cells in H&E-stained tissue and collagen deposition by Masson’s trichrome staining. **(J)** Quantitative analysis of the mean density of TGF-β1 and α-SMA expression in the wounds **(C,D)**. Longitudinal healing rates and macroscopic scores were analyzed by two-way repeated measures ANOVA with Bonferroni *post hoc* correction **(I,J)**. Multi-group comparisons were analyzed by one-way ANOVA with Tukey’s *post hoc* test. Data are presented as mean ± SD. *p < 0.05, **p < 0.01, ***p < 0.001 and ****p < 0.0001.

To validate our *in silico* predictions, an OU rat model was used. After local administration of hPDLSCs on post-injury day 1 (D1), we performed longitudinal monitoring of the wound bed and histological assessment using H&E and Masson’s trichrome staining (Figure 4A).

Macroscopic and microscopic evaluations confirmed that hPDLSC-treated ulcers healed significantly faster than those in the untreated model group (Figure 4B). Specifically, by day 2, the control group exhibited persistent edema and thick pseudomembrane formation, whereas the hPDLSC group showed attenuated inflammation and reduced swelling. By day 4, the ulcerated area in the treatment group had shrunk markedly, with nascent mucosal epithelium progressively replacing the pseudomembrane. These findings suggest that hPDLSCs accelerate the transition from the inflammatory phase to re-epithelialization, resulting in faster wound closure.

On day 6, the ulcer healing rate in the hPDLSCs group reached 80%, compared with 50% in the model group. On day 8, ulcers in the hPDLSCs group were almost completely healed (healing rate of 97%), with smooth, flat mucosa and no scarring. In contrast, the healed area in the model group was only 70%, and the wound remained swollen and inflamed (Figure 4C). These observations indicate that hPDLSCs effectively promoted ulcer repair. According to the objective ulcer grading criteria, healing scores also showed that the quality of healing in the hPDLSCs group was superior to that of the model group (Figure 4D).

Next, we performed histologic analyses to assess neocapillary formation, collagen deposition, fibroblast proliferation, and inflammatory cell infiltration, which are widely used as markers of wound healing (Figure 4E). HE staining showed that the hPDLSCs group rats had fewer inflammatory cells, more neocapillaries, earlier epithelial crawling, and eventual wound closure. By contrast, the model group rats had more neutrophils and lymphocytes, fewer new capillaries, and larger wounds. Fibroblasts in the lamina propria of the hPDLSCs group were regularly arranged, parallel to the epithelium, and sparse. In the model group, fibroblasts were disorganized and more numerous. Masson staining was additionally used to visualize collagen accumulation in the tissues, revealing fewer collagen fibers in the rats from the hPDLSCs group.

To show that injected hPDLSCs can survive within the oral mucosa of OU rats and fulfill specific functions, we used FISH to detect hPDLSC presence in the oral mucosa. PLAP-1, a marker for the periodontal ligament lineage, including stem and progenitor cells, was employed for labeling. In the hPDLSC group, the injected cells were positive for PLAP-1, while the model group showed no such expression (Figure 4F). These results indicate that the injected hPDLSCs can survive in the oral mucosa of OU rats. Figure 3G shows angiogenesis in the oral ulcer region of SD rats on Day 8, with the hPDLSC group displaying a higher number of newly formed blood vessels (indicated by green arrows). Inflammatory cell counts in the corresponding regions (Figure 4I) showed that the hPDLSC group had significantly fewer inflammatory cells at the ulcer site than the Model group on both Day 4 and Day 8. Quantitative assessment of collagen fiber deposition further revealed a markedly higher density in hPDLSC-treated ulcers relative to the Model group at both time points. IHC results (Figure 4H) indicated that expression levels of TGF-β1 and α-SMA were significantly reduced in the ulcer tissues treated with hPDLSCs compared to the Model group on D4 and D8, consistent with the WB findings. These results indicate that hPDLSCs reduce both inflammation and fibrosis in SD rat OU. Semi-quantitative statistical analysis of the IHC data is provided in Figure 4J.

### 3.4 RNA-seq reveals that hPDLSCs downregulate TGF-β and ERK signaling pathways in ulcer healing

**FIGURE 5:**
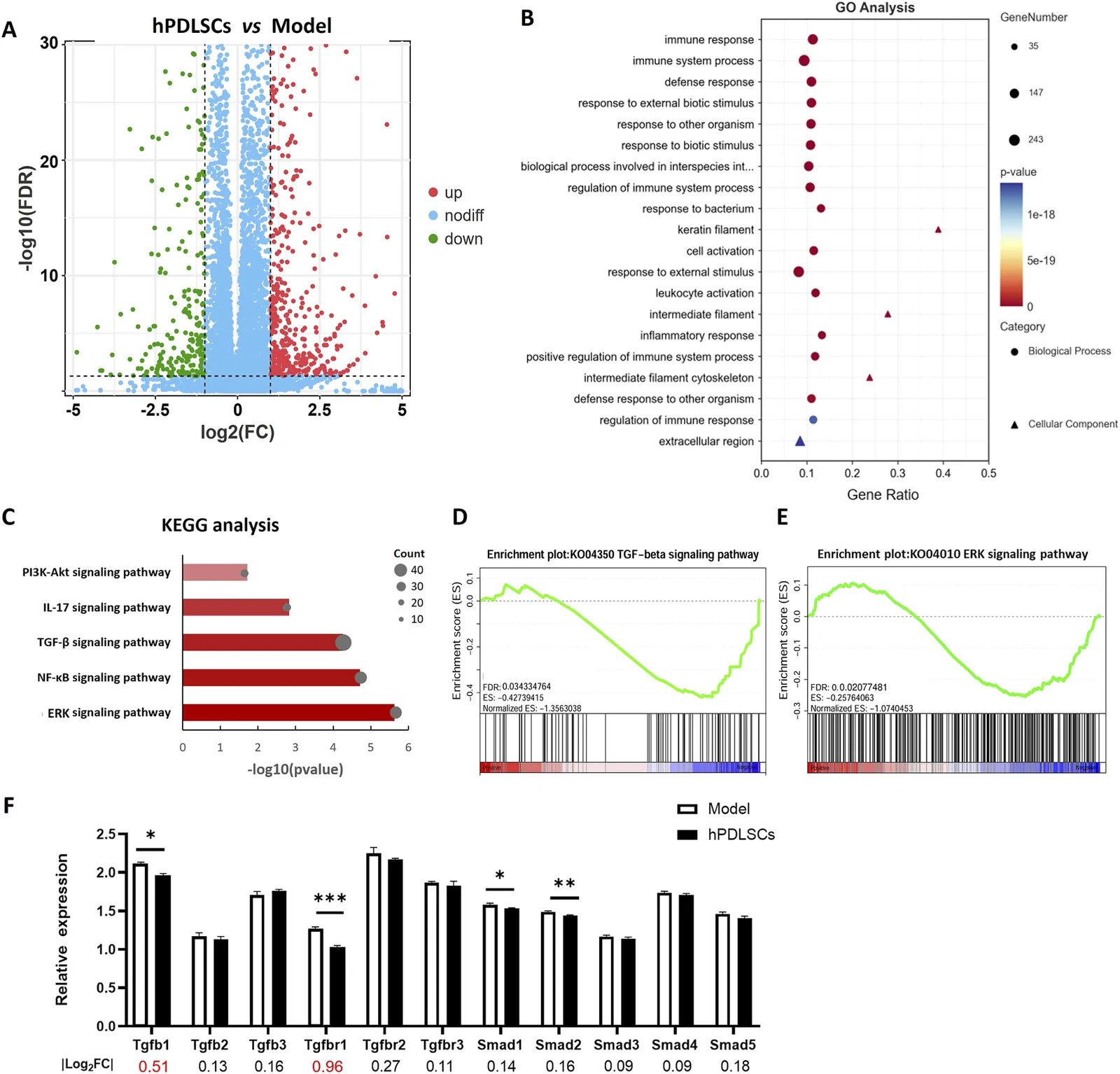
Transcriptomic profiling of oral ulcers treated with hPDLSCs via RNA-sequencing. **(A)** Volcano plot of DEGs. **(B)** GO enrichment of DEGs. **(C)** KEGG pathway of DEGs. **(D)** GSEA of TGF-β genes. **(E)** GSEA of ERK genes. **(F)** Expression levels of relevant genes in TGF-β enrichment pathway.

To explore the molecular mechanism by which hPDLSCs facilitate the healing of oral mucosa ulcers in OU rats, RNA-seq analysis was conducted on samples from these rats. This revealed 1,002 DEGs, including 631 upregulated and 371 downregulated genes, as illustrated in the volcano plot (Figure 5A).

GO analysis of all DEGs revealed notable changes in BP terms related to immune response, defense mechanisms, and reactions to external biotic factors. Changes in CCs for keratin filament and intermediate filament were also observed (Figure 5B). This suggests that DEGs are primarily involved in inflammatory response and epithelial repair during the oral ulcer healing process.

KEGG pathway analysis of all DEGs identified several significant pathways, with the top five being the ERK signaling pathway, NF-κB signaling pathway, TGF-β signaling pathway, IL-17 signaling pathway, and PI3K-Akt signaling pathway (Figure 5C). All of these pathways play critical roles in inflammation, immunomodulation, and tissue repair, suggesting that hPDLSC-treated DEGs have an important role in regulating the above biological processes (Masoomikarimi and Salehi, 2022). GSEA confirmed that hPDLSCs significantly downregulated the TGF-β signaling pathway and the ERK pathway, as both pathways exhibited negative normalized enrichment scores (NES) (Figures 5D–F).

### 3.5 hPDLSCs alleviate oral ulcer fibrosis and inflammation via TGF-β1/smad and ERK pathway suppression

Transcriptomic and molecular analyses indicated that inhibition of the TGF-β1/Smad and ERK pathways represents one key mechanism by which hPDLSCs exert anti-fibrotic and anti-inflammatory effects.

**FIGURE 6:**
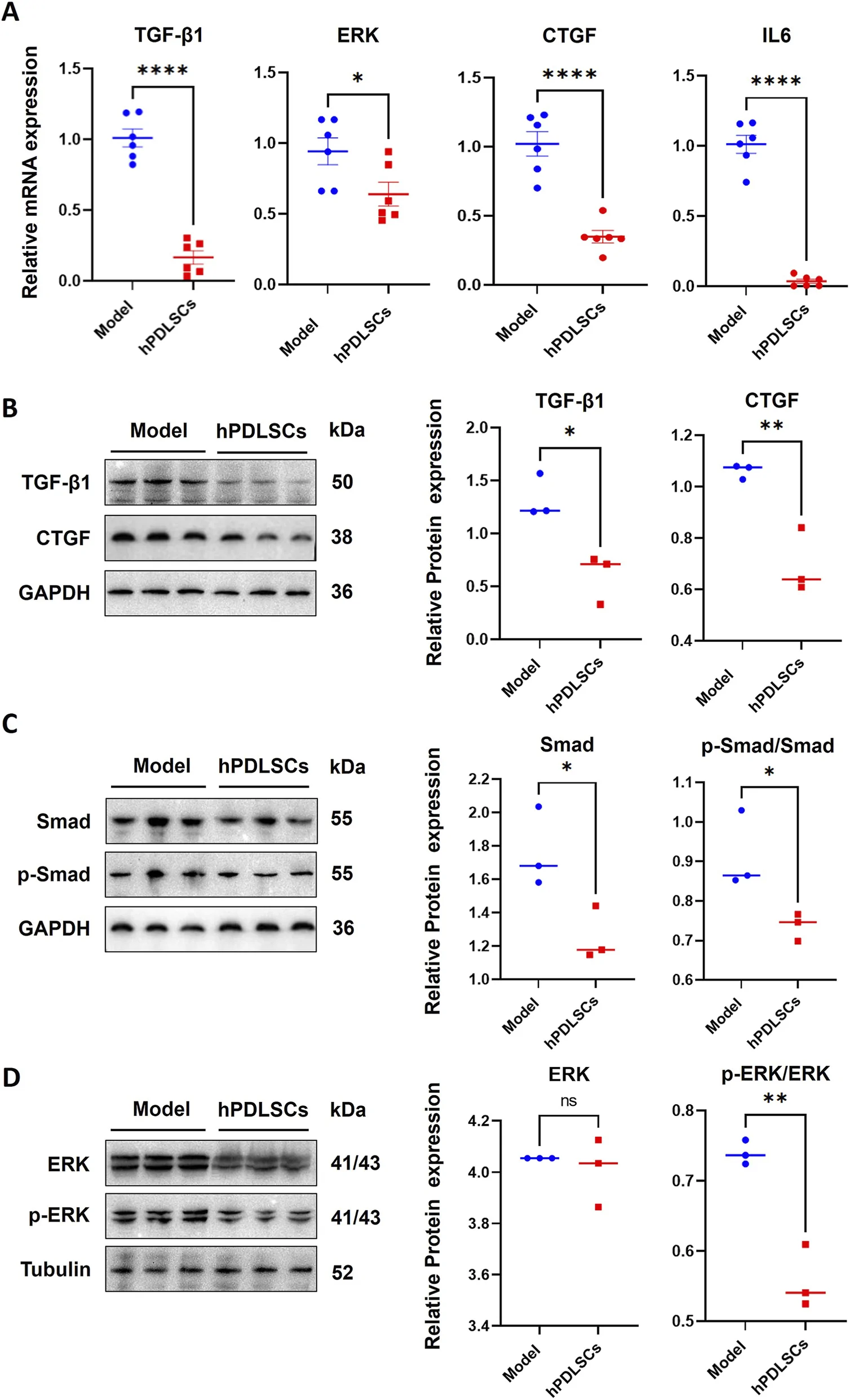
hPDLSCs treatment inhibits the TGF-β1/Smad and ERK pathways, reducing inflammation and fibrosis. **(A)** RT-qPCR of TGF-β1, ERK, CTGF, and IL-6 mRNA (n = 6, mean ± SD). **(B)** WB for TGF-β1 and CTGF in OU rats. **(C)** WB for Smad and p-Smad. **(D)** WB for ERK and p-ERK. Data are presented as mean ± SD. Two-group comparisons (Model *vs*. hPDLSCs) were performed using unpaired two-tailed Student’s t-test. *p < 0.05, **p < 0.01, ***p < 0.001 and ****p < 0.0001 vs Model group.

Validation of the RNA-Seq results showed that hPDLSCs reduced the expression of genes and proteins associated with the TGF-β1/Smad and ERK signaling pathways. Specifically, in analyzing mRNA levels of fibrosis markers and inflammatory indicators in oral mucosa, the expression of CTGF and IL-6 was decreased after treatment with hPDLSCs. Additionally, RT-qPCR demonstrated reduced expression of TGF-β1 and ERK in OU rats treated with hPDLSCs (Figure 6A).

WB results further showed that the protein levels of TGF-β1, CTGF, Smad, and p-Smad were decreased in the hPDLSCs group (Figures 6B,C). To further investigate which factors secreted by hPDLSCs mediate the regulation of TGF-β1, we detected the cytokines in the supernatant of hPDLSC culture medium and found that IL-8 was the only cytokine significantly upregulated (Supplementary Figure S1A). IL-8 has been reported to recruit neutrophils in the inflammatory microenvironment, which is consistent with our previous experimental findings. We therefore hypothesize that hPDLSCs secrete IL-8 to decrease fibrosis during oral ulcer healing by inhibiting the TGF-β1/Smad signaling pathway.

Meanwhile, WB also showed that protein levels of genes related to the ERK signaling pathway were further reduced in the hPDLSCs group (Figure 6D). These results suggested that hPDLSCs inhibited the ERK pathway. The ERK pathway is part of the inflammatory pathway. ERK is a key component of the MAPK signaling pathway, which is involved in inflammatory responses and the regulation of cell growth, among other biological processes (Bora and Yaba, 2021; Zhang et al., 2021).

### 3.6 IL-8 secreted by hPDLSCs suppresses TGF-β1 expression in oral epithelial cells via a paracrine mechanism

To test this hypothesis, we established five experimental groups using a Transwell co-culture system (Supplementary Figure S1B): ① HOK alone; ② hPDLSCs + HOK; ③ HOK + neutrophils; ④ hPDLSCs + HOK + neutrophils; and ⑤ hPDLSCs + HOK + neutrophils + IL-8 neutralizing antibody. As shown in Supplementary Figure S1C, HOK cells cultured alone (Group 1) expressed TGF-β1 at a moderate basal level. Co-culture of hPDLSCs with HOK (Group 2) did not substantially alter TGF-β1 expression compared to Group 1. Addition of neutrophils (Group 3) markedly elevated TGF-β1 expression above basal levels, consistent with an inflammatory response. The inclusion of hPDLSCs in the presence of neutrophils (Group 4) reduced TGF-β1 expression to a level below that of both Group 1 and Group 3. Neutralization of IL-8 (Group 5) reversed this suppression, restoring TGF-β1 expression to a moderate level comparable to that of Group 1 and Group 2. These results demonstrate that, under inflammatory conditions, hPDLSCs suppress TGF-β1 expression in oral epithelial cells via an IL-8-dependent paracrine mechanism, providing functional evidence for the anti-fibrotic effect of hPDLSCs.

## 4 Discussion

OU are characterized by localized mucosal necrosis, pain, and swelling (Fitzpatrick et al., 2019; Griffin et al., 2022). While the oral mucosa typically heals regeneratively without scarring, systemic comorbidities—such as diabetes or autoimmune disorders—can shift this process toward pathological scarring through persistent inflammation and aberrant collagen deposition, potentially impairing oral functions such as mastication and speech (Turabelidze et al., 2014; Noor et al., 2021; Pereira and Sequeira, 2021; Pan et al., 2024).

The orchestration of mucosal repair involves a precise sequence of hemostasis, inflammation, proliferation, and remodeling (Pereira and Sequeira, 2021; Ngeow et al., 2022). A key determinant in this process is the macrophage polarization shift: whereas M1 phenotypes drive initial defense via pro-inflammatory cytokines (IL-1, IL-6, TNF-α), M2 phenotypes facilitate resolution through anti-inflammatory factors (IL-10, VEGF, TGF-β) (Sindrilaru et al., 2011; Lin et al., 2022). However, TGF-β1—a multifunctional growth factor—is a double-edged sword. Its overexpress-sion is strongly associated with excessive extracellular matrix (ECM) accumulation and myofibroblast activation, leading to mucosal fibrosis (Qin et al., 2023; Pan et al., 2024). We emphasize that hPDLSCs inhibit the pathological sustained phase of TGF-β1/Smad signaling, not its acute physiological functions in early wound healing. Although various stem cell therapies have been shown to accelerate OU closure, their ability to achieve scar-free healing has remained underexplored (Zhou et al., 2023; Chen et al., 2025).

In this study, we used hPDLSCs, which we found to be highly sensitive to TGF-β1 signaling because of their origin in periodontal tissues, where the TGF-β1 superfamily is actively involved in homeostasis. Through integrated GEO-based bioinformatics (identifying 92 key genes) and RNA-seq analysis, we pinpointed the TGF-β1/Smad and ERK1/2 cascades as the primary regulatory axes for hPDLSC-mediated repair.

Our *in vivo* rat model showed that local hPDLSC injection not only significantly increased the healing rate—achieving complete epithelialization by day 8—but also fundamentally altered the quality of repair. Histologically, hPDLSC-treated tissues showed reduced inflammatory infiltration, accelerated epithelial migration, and remarkably well-organized, parallel fibroblast alignment, in contrast to the disorganized collagen deposition in the control group. Mechanistically, the observed anti-inflammatory and anti-fibrotic effects of hPDLSCs were associated with downregulation of the TGF-β1/Smad/CTGF signaling axis. Furthermore, our findings revealed that hPDLSCs concurrently modulate the ERK signaling pathway, which influences fibroblast proliferation and late-stage tissue remodeling. By simultaneously inhibiting both the TGF-β1/Smad and ERK pathways, hPDLSCs achieve multi-targeted regulation of the wound microenvironment. This study indicate that hPDLSC-mediated suppression of TGF-β1 and CTGF/α-SMA signaling aligns with prior findings that reducing TGF-β attenuates fibrosis (Kalahasti et al., 2025). It has been reported that hPDLSCs may also activate alternative regenerative pathways in addition to suppressing TGF-β and ERK signalling. For instance, the NF-κB pathway has been implicated in hPDLSC-mediated diabetic wound repair (Cong et al., 2025), and the Wnt signalling cascade is known to accelerate re-epithelialization and neovascularization in MSC-based therapies (Park et al., 2026). Further studies are required to confirm whether these pathways contribute to the enhanced oral ulcer healing observed in the present model.

Despite these insights, several limitations remain regarding translational application. First, the acute rat model used here does not fully recapitulate the complex microenvironment of chronic oral ulcers; the primary goal of this acute model was to establish a baseline mechanistic understanding, and future studies in chronic models are necessary to validate and extend our findings. Second, direct causal validation of the TGF-β1/Smad and ERK pathways using pathway-specific inhibitors or genetic manipulation has not been performed. We have provided *in vitro* functional evidence showing that hPDLSCs suppress TGF-β1 in HOK cells via an IL-8-dependent mechanism. However, direct causal validation of the intracellular TGF-β1/Smad and ERK pathways using pathway-specific inhibitors or genetic manipulation has not been performed, and this remains a limitation for future studies. The anatomical and immunological differences between rodents and humans necessitate further validation in larger animal models. Future research must assess long-term safety and stability, as well as mechanistic rescue experiments, to definitively confirm the clinical potential of hPDLSCs for oral ulcer healing.

## 5 Conclusion

In summary, our findings highlight the role of the TGF-β1/Smad signaling pathway in the treatment of OU with hPDLSCs. Our findings suggest that hPDLSCs attenuate oral ulcer fibrosis and inflammation, at least in part, through modulation of the TGF-β1/Smad and ERK signaling pathways. While this study provides mechanistic insights, direct causal validation through rescue experiments is needed to confirm the therapeutic potential of hPDLSCs for oral ulcer healing.

## Data Availability

The datasets presented in this study can be found in online repositories. The names of the repository/repositories and accession number(s) can be found in the article/Supplementary Material.
