# Supplementary material for: Human periodontal ligament stem cells promote oral ulcer healing in rats through modulation of TGF-β1/smad signaling
Source: Front Cell Dev Biol. 2026 Aug 28;14:1824785. doi: 10.3389/fcell.2026.1824785 (PMC13562027; doi:10.3389/fcell.2026.1824785)
Supplement: Supplementary file 1 [file Supplementaryfile1.pdf]

Supplementary Material

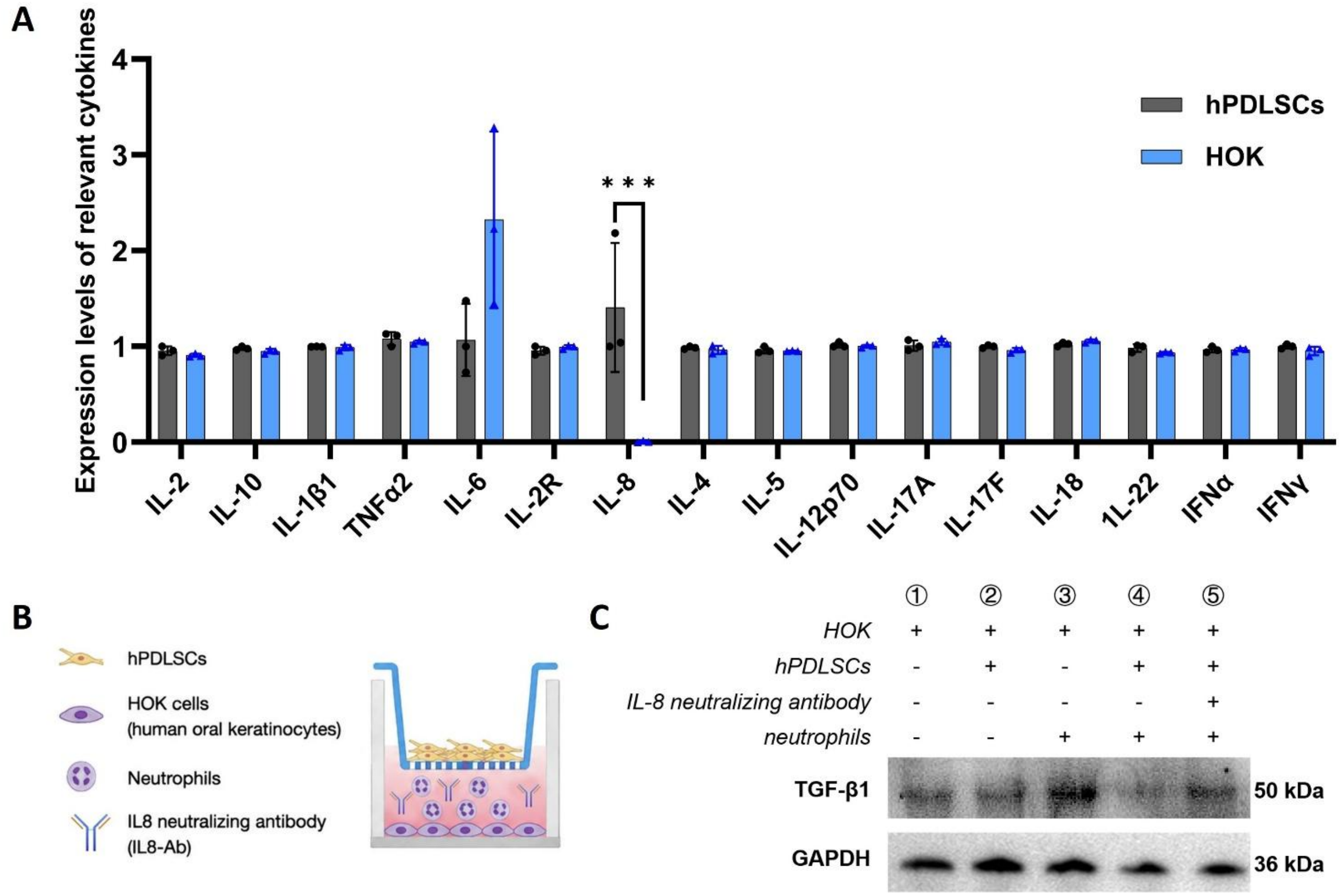

Supplementary Figure 1 (**A**) Cytokine secretion profiles in culture supernatants of hPDLSCs and HOK. Supernatants were collected after 48 h of culture, and cytokine levels were measured using a chemiluminescence immunoassay. Among the detected cytokines, interleukin-8 (IL-8) was identified as a candidate factor highly expressed in hPDLSC supernatants compared to HOK supernatants. Data are presented as mean ± SD (n = 3). $^{***}p < 0.001$ *vs*. HOK group; (**B**) Schematic illustration of the Transwell coculture system for evaluating the regulatory effects of IL8-Ab on hPDLSCs under different coculture conditions; (**C**) Western blot analysis of TGF-β1 expression in HOK under various coculture conditions.
